# Effect of temperature, nutrients and growth rate on picophytoplankton cell size across the Atlantic Ocean

**DOI:** 10.1038/s41598-024-78951-w

**Published:** 2024-11-14

**Authors:** Emilio Marañón, Cristina Fernández-González, Glen A. Tarran

**Affiliations:** 1 Centro de Investigación Marina and Facultad de Ciencias del Mar, Universidade de Vigo, Vigo, Spain; 2 Plymouth Marine Laboratory, Plymouth, UK

**Keywords:** Ecology, Microbiology, Ecology, Environmental sciences, Ocean sciences

## Abstract

The cell size of picophytoplankton populations affects their ecology and biogeochemical role, but how different environmental drivers control its variability is still not well understood. To gain insight into the role of temperature and nutrient availability as determinants of picophytoplankton population mean cell size, we carried out five microcosm experiments across the Atlantic Ocean (45°N-27°S) in which surface plankton assemblages were incubated under all combinations of three temperatures (in situ, 3 °C cooling and 3 °C warming) and two nutrient levels (unamended and addition of nitrogen and phosphorus). The overall range of variability in cell volume was 5-fold for *Prochlorococcus*, 8-fold for *Synechococcus* and 6-fold for the picoeukaryotes. We observed, in all the treatments and in the control, a consistent trend toward larger mean cell sizes over time for both *Prochlorococcus* and *Synechococcus*, which was likely the result of sample confinement. Changes in temperature and nutrient status alone did not cause clear changes in cell size, relative to the control, but the combination of warming and nutrient addition resulted in an increase in *Prochlorococcus* and *Synechococcus* cell size. The largest increases in cell volume were associated with slow or negative population net growth rates. Our results emphasize the importance of considering changes in biovolume to obtain accurate estimates of picophytoplankton biomass and suggest that the inverse relationship between growth rate and population mean cell size may be a general pattern in marine phytoplankton.

## Introduction

Picophytoplankton are photosynthetic unicells with a cell diameter ≤ 2 μm that include the cyanobacteria *Prochlorococcus* and *Synechococcus* and the phylogenetically diverse picoeukaryotes^1,2^, collectively contributing between one quarter and half of total marine primary production^3,4^. While the role of picophytoplankton is particularly noticeable in oligotrophic regions of the tropical and subtropical open ocean, where they sustain > 60% of total primary productivity^5,6^, this group of photoautrophs can also represent a substantial (> 30%) fraction of phytoplankton standing stocks in coastal, productive ecosystems during part of the year^7,8^. Despite their small cell size and comparatively slow sinking rates, picophytoplankton also drive the biological carbon pump, through both direct and indirect pathways, with several studies suggesting that their global contribution to the export flux of biogenic carbon from the euphotic zone is similar to their share of total net primary production^4,9^. In view of their wide ecological and biogeochemical significance, there is an increasing need to ascertain how different environmental drivers, including temperature and nutrient availability, may affect the functional traits of picophytoplankton and their contribution to community and ecosystem processes^10–12^.

Cell size affects multiple aspects of the physiology, ecology and biogeochemical role of phytoplankton, including growth and metabolic rates, elemental stoichiometry, light and nutrient acquisition, mortality due to predation and viral lysis, and sinking out of the euphotic zone^13–17^. For example, within picophytoplankton, population mean cell size is correlated with maximum growth rate and the ability to cope with extremely low nutrient availability or to exploit transient nutrient inputs. The smallest picophytoplankton, *Prochlorococcus* (ca. 0.6 μm in equivalent spherical diameter, ESD), has slow maximum growth rates but is highly adapted to ultraoligotrophic conditions, where it dominates picophytoplankton biomass^18^, whereas the slightly larger *Synechococcus* (ESD 1 μm) can sustain faster growth rates and increases its abundance when nutrient availability is enhanced^19,20^. Picoeukaryotes, with a mean ESD of ca. 2 μm, present lower abundances than picocyanobacteria do but, owing to their higher maximum growth rates and maximum nutrient uptake rates, are often the dominant picophytoplankton group in nutrient-rich environments^2,21,22^. Despite the well-recognized importance of cell size, most picophytoplankton studies have focused solely on the abundance of each group, without reporting their mean population cell size. Indeed, Buitenhuis et al. (2012) emphasized the need for routine measurements of picophytoplankton cell size, based on the flow cytometry scattering signal, as a prerequisite not only to obtain accurate estimates of biomass but also to increase our knowledge of the variability and controlling factors of this critical functional trait.

Picophytoplankton cell size changes widely across multiple scales of spatial and temporal variability, including vertically over the euphotic zone^23–26^, geographically across regions and with latitude^23,25,27^ and temporally over diel^28,29^ and seasonal cycles^24,26,30^. While some trends appear to be consistent among studies, such as the increase in the mean cell size of *Prochlorococcus* and *Synechococcus* with increasing depth in the euphotic zone^1,23,24,26,31^, there are discrepancies in other patterns. For instance, the cell size of small picoeukaryotes has been found to increase with depth in some studies^1,24^ but not in others^32,33^. The interpretation of these in situ observations, in terms of the underlying mechanisms, is hindered by the fact that key environmental drivers tend to covary in the sea. Thus, it is not immediately clear if the increase in cell size of *Prochlorococcus* and *Synechococcus* near the base of the euphotic layer results from colder temperatures, decreased irradiance, enhanced nutrient supply, or a combination of these or other factors. Experimental approaches, in which temperature, irradiance and nutrient availability can be controlled, are needed to disentangle and quantify the effect of these variables on cell size. More generally, manipulative experiments are expected to play an increasingly central role in the search for mechanistic understanding of the biological impacts of multiple drivers associated with global change^34,35^.

In addition to the effect of temperature and nutrients on cell size, there is growing evidence that changes in growth rates can also play a role. Results from chemostat experiments, in which *Synechococcus* populations were maintained at fixed growth rates over a range of temperatures, indicated that temperature had no effect on cell size estimated as carbon biomass^36^. Recently, a study with multiple *Synechococcus* strains grown at various temperatures failed to find a relationship between temperature and cell size, but reported an inverse relationship between growth rate and cell size^37^. Furthermore, the association between decreased cell size during the exponential growth phase and increased cell size during the stationary phase in batch cultures has been reported both for picophytoplankton^38^ and for large eukaryotic algae including diatoms, dinoflagellates and chlorophytes^39,40^. There is, however, a paucity of experimental studies with natural communities that address the changes in phytoplankton cell size in response to temperature and nutrient availability, while also considering the effects of growth rate.

During October-November 2019 we conducted five microcosm experiments with natural plankton assemblages across the Atlantic Ocean (45°N-27°S), in which changes in picophytoplankton cell size were monitored under all combinations of three temperatures (in situ, 3 °C warming and 3 °C cooling) and two nutrient treatments (unamended and enrichment with nitrogen and phosphorus). In a previous study, we described the effects of temperature and nutrient supply on phytoplankton abundance, chlorophyll biomass and cell-specific light-harvesting capacity^38^. Here we report on the changes in the mean population cell size of *Prochlorococcus*, *Synechococcus* and the picoeukaryotes in response to changes in temperature and nutrients and investigate the relationship between population net growth rates and mean cell size. Our observations allow us to characterize the variability in picophytoplankton cell size as a function of controlled growth conditions, thus helping to constrain abundance-based biomass estimates, and give insight into the role of temperature, nutrients and growth rate as drivers of cell biovolume.

## Materials and methods

### Sampling and experimental setup

We conducted five on-deck incubation experiments during the cruise Atlantic Meridional Transect (AMT) 29, which took place between Southampton (UK) and Punta Arenas (Chile) during October-November 2019. The experiments were conducted with intact microbial plankton assemblages, which include picocyanobacteria, photosynthetic and mixotrophic eukaryotes, heterotrophic bacteria and microzooplankton grazers. A full description of the sampling and experimental procedures is given elsewhere^38^. Briefly, surface seawater (5–7 m depth) was collected just before dawn using Niskin bottles attached to a rosette equipped with a CTD probe (SeaBird, SBE, 911plus/917) and a chlorophyll fluorescence sensor. The location and date of sampling for the incubation experiments were as follows: 44.7°N, 16.2°W, 19 October; 28.8°N, 33.0°W, 19 October; 12.7°N, 28.5°W, 1 November; 7.4°S, 25.0°W, 7 November; and 26.7°S, 26.7°W, 13 November 2019. At each location, unfiltered seawater from the Niskin bottles was transferred into a single 20 L, HCl-washed, polycarbonate carboy (Nalgene), from which 18 HCl-washed, 1 L polycarbonate bottles were filled. Nine bottles were supplemented with ammonium nitrate (NH_4_NO_3_) and sodium dihydrogen phosphate (NaH_2_PO_4_) to increase the concentration of nitrate and ammonium by 1 µmol L^− 1^ and the concentration of phosphate by 0.2 µmol L^− 1^, while the remaining bottles were left unamended. We then placed six bottles (3 nutrient-amended and 3 non-amended bottles) inside each tank of an on-deck temperature-controlled incubator. The temperatures in the three tanks were set as follows: in situ (sea surface) temperature, in situ temperature −3 °C, and in situ temperature + 3 °C. The temperature inside each tank was monitored continuously by the incubator’s internal temperature-controlling system as well as by TinyTag Aquatic 2 dataloggers that recorded the temperature every 2 min. All incubation bottles were covered by a neutral density mesh which allowed 70% of photosynthetically active radiation (PAR) to pass through. Given the values of the PAR vertical extinction coefficient typically measured in the tropical and subtropical open ocean (0.03–0.06 d^− 1^), phytoplankton populations from 5 to 7 m depth are expected to receive between 65 and 85% of incident PAR. Immediately after nutrient amendment, samples for flow cytometry analysis were taken from all the bottles (t = 0), which were sampled every day before dawn until the experiments were terminated after 96 h of incubation (t = 4).

### Chlorophyll *a* and inorganic nutrient concentration

For the determination of initial chlorophyll *a* concentration (Chl *a*), samples (250 mL in volume) were collected in triplicate from the 20 L carboy and filtered through 0.2 μm polycarbonate filters, which were kept frozen at − 20 °C until analysis. We measured Chl *a* fluorometrically, after filter extraction in 90% acetone at 5 °C overnight, using a Trilogy fluorometer (Turner Designs). To determine the concentration of dissolved inorganic nutrients, 60 mL samples from 8 to 10 depths in the upper 200 m were collected into acid-washed high-density polyethylene HDPE (Nalgene) bottles, which were kept frozen at − 20 °C until analysis in the laboratory on land. The concentrations of nitrate, phosphate and silicate were determined using a SEAL analytical AAIII segmented-flow, colorimetric nutrient auto-analyzer. We calculated the depth of the nitracline at each station as the first depth at which the concentration of nitrate was greater than 0.5 µmol L^− 1^. The depth of the deep chlorophyll maximum (DCM) at each station was taken as the depth at which chlorophyll fluorescence peaked in the CTD profiles.

### Flow cytometry analyses

Samples were taken in 6-mL Falcon tubes and kept in the dark at 4 °C until analysis, within 2 h of sampling, by flow cytometry following the methods described before^41^. Briefly, cell abundance and optical properties were measured with a Becton Dickinson FACSortTM flow cytometer (BD Biosciences) equipped with an air-cooled laser delivering blue light at 488 nm. In addition to measuring the cell abundance, the flow cytometer also measured chlorophyll red fluorescence (> 650 nm), phycoerythrin fluorescence (585 ± 21 nm), and side scatter (SSC), which is the light scattered at 90° to the direction of the laser beam. Light scatter and fluorescence data were processed with CellQuest software (Becton Dickinson, Oxford). Scatter plots of SSC vs. orange fluorescence were used to identify and enumerate *Synechococcus*, while plots of SSC vs. red fluorescence (without *Synechococcus*) were used to count *Prochlorococcus* and picoeukaryotic algae.

### Estimates of cell diameter

**Fig. 1:**
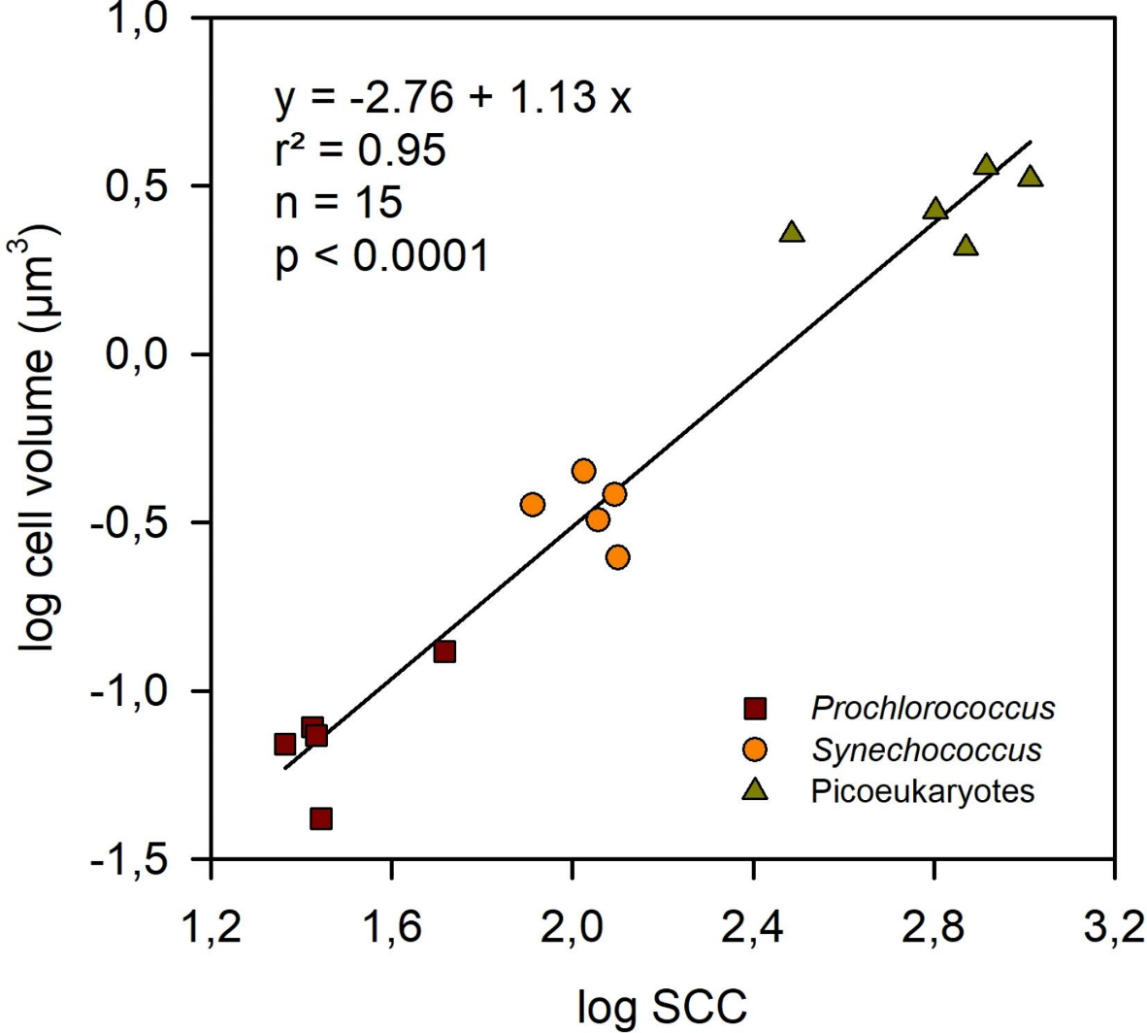
Log-log relationship between side scattering (SSC) and cell biovolume for *Prochlorococcus*, *Synechococcus* and picoeukaryotes. The linear regression model shown was used to calculate the cell biovolume from the SSC data for each picophytoplankton group in all the experiments.

We conducted several size-fractionation experiments to estimate the mean cell diameter of *Prochlorococcus*, *Synechococcus* and picoeukaryotic algae. The approach involved passing samples of seawater through membrane filters (Whatman NucleporeTM polycarbonate filters of 47 mm diameter) from 0.2 to 3 μm and analyzing the resulting filtrates by flow cytometry. The filters were pre-wetted with 0.2 μm filtered seawater and then placed in in-line polycarbonate filter housings clamped onto a burette stand with a 5 mL flow cytometry tube below to collect the filtrate. Five-millilitre syringes without pistons were placed in the filter housing inlet, and 4 mL of seawater were then pipetted into the syringe and allowed to gravity filter through the chosen filter. The resulting filtrates were analyzed by flow cytometry and the number of cells per sample of the different groups was recorded. The percentage of cells remaining in each filtrate, relative to those in unfiltered water, was plotted against filter pore size to produce curves as shown in the example in Fig. S1. For each plankton group, a line was drawn from the 50% mark on the Y axis. Where it intersected with the curve a vertical line was drawn down to the X axis and the point of intersection with the X axis was recorded as the median cell size for that group. The cell volume was calculated from the estimated cell diameter by assuming that the cells were spherical. We then plotted, using log_10_-transformed data, the cell volume calculated for each population as a function of its SSC value measured on the same sample (Fig. 1). The resulting linear regression equation was used to convert SSC to cell volume (V, µm^3^):

$${\text{log V }}= - {\text{2}}.{\text{765}}\,+\,{\text{1}}.{\text{127}} \times {\text{log SSC }}({{\text{r}}^{\text{2}}}\,=\,0.{\text{95}},n\,=\,{\text{15}},p\,<\,0.000{\text{1}})$$

### Data analysis and statistics

The population-based growth rates of each picophytoplankton group were calculated for days 2–4 as ln(N_2_/N_1_)/Δt, where N_1_ and N_2_ are the cell abundances measured on days 2 and 4, respectively, and Δt is the time interval elapsed (2 days). We concentrated on days 2–4 because during this period changes in cell abundance and chlorophyll *a* concentration in response to the various treatments became more noticeable^38^. We also calculated biovolume-based growth rates for the same period (days 2–4) as ln(B_2_/B_1_)/Δt, where B_1_ and B_2_ are the biovolume concentrations (product of cell abundance and cell volume) measured on days 2 and 4, respectively. Changes in picophytoplankton cell volume in response to the different treatments during each experiment were assessed in two ways. First, linear regression analysis was used to determine the slope and significance of the relationship between cell volume (dependent variable, *y*) and sampling time (independent variable, *x*). Second, we calculated the quotient between the mean cell volume measured on days 3 and 4 and the initial cell volume measured at t = 0. To evaluate the effect of each experimental treatment on cell volume, we calculated the treatment to control cell volume ratio, which is the mean cell volume measured under a given treatment divided by the mean cell volume measured in the control (in situ temperature, no nutrient addition). The statistical significance of the differences between cell volume in each treatment and in the control was assessed with the Student’s t-test using log-transformed data. All the statistical analyses were performed with IBM SPSS Statistics 24.

## Results

### Initial conditions

The first experiment (44.7°N) was conducted in temperate, mesotrophic waters with a sea surface temperature (SST) of 17.6 °C and a surface chlorophyll *a* concentration (Chl *a*) of 0.36 µg L^− 1^, whereas the remaining four experiments (28.8°N – 26.8°S) took place in tropical and subtropical locations, all characterized by warm SSTs (≥ 25 °C), low Chl *a* (≤ 0.25 µg L^− 1^) and low nitrate concentration (≤ 0.05 µmol L^− 1^) (Table 1). Within these four tropical and subtropical stations, the degree of oligotrophy differed markedly, as reflected in metrics such as surface Chl *a*, nitracline depth and depth of the deep chlorophyll maximum (DCM). Thus, the stations at 28.8°N and 26.8°S were the most oligotrophic, with very deep nitraclines (128–146 m) and DCM (115–120 m) and low surface Chl *a* (0.09 µg L^− 1^). In contrast, the station at 12.7°N, located in a region influenced by the equatorial upwelling, was the least oligotrophic, having a much shallower nitracline (15 m) and DCM (43 m) as well as higher Chl *a* (0.25 µg L^− 1^). The initial abundances of *Prochlorococcus* and *Synechococcus* were more variable than those of the picoeukaryotes (Table 1). The highest abundances were measured at 12.7°N for all the groups (550,000, 35,000 and 3,800 cell mL^− 1^ for *Prochlorococcus*, *Synechococcus* and picoeukaryotes, respectively). The lowest picocyanobacterial abundances were measured at 44.7°N (61,000 and 1,400 cell mL^− 1^ for *Prochlorococcus* and *Synechococcus*, respectively), whereas the lowest picoeukaryote abundance (950 cell mL^− 1^) was found at 28.8°N. The range of variability in the mean cell volume across locations was wider for picoeukaryotes (1.1–4.3 μm^3^) than for *Prochlorococcus* (0.07–0.15 μm^3^) and *Synechococcus* (0.3–0.6 μm^3^).

**Table 1:** Initial physico-chemical and biological properties of the surface seawater used to conduct onboard experiments at five locations during the AMT29 cruise. The nitracline depth and the depth of the deep chlorophyll maximum (DCM) at each station are also shown.

Latitude	44.7°*N*	28.8°*N*	12.7°*N*	7.4°S	26.8°S
Sea surface temperature (°C)	17.6	25.5	28.4	25.5	25.5
NO_3_^−^ concentration (µmol L^− 1^)	0.16	0.02	0.05	0.02	0.02
HPO_4_^2−^ concentration (µmol L^− 1^)	0.02	0.02	0.02	0.12	0.07
H_4_SiO_4_ concentration (µmol L^− 1^)	0.92	1.16	1.84	1.13	1.29
Chl *a* concentration (µg L^− 1^)	0.36	0.09	0.25	0.13	0.09
Nitracline depth (m)	60	146	15	94	128
DCM depth (m)	58	115	43	86	120
*Prochlorococcus* abundance (cell mL^− 1^)	61,527	95,545	550,007	242,061	198,072
*Prochlorococcus* cell volume (µm^3^)	0.15	0.07	0.12	0.07	0.06
*Synechococcus* abundance (cell mL^− 1^)	1,400	1,648	35,018	2,057	3,372
*Synechococcus* cell volume (µm^3^)	0.33	0.40	0.57	0.39	0.36
Picoeukaryote abundance (cell mL^− 1^)	1,999	947	3,823	1,775	1,966
Picoeukaryote cell volume (µm^3^)	1.09	4.27	4.05	2.48	2.95

### Temporal variability of cell volume

**Fig. 2:**
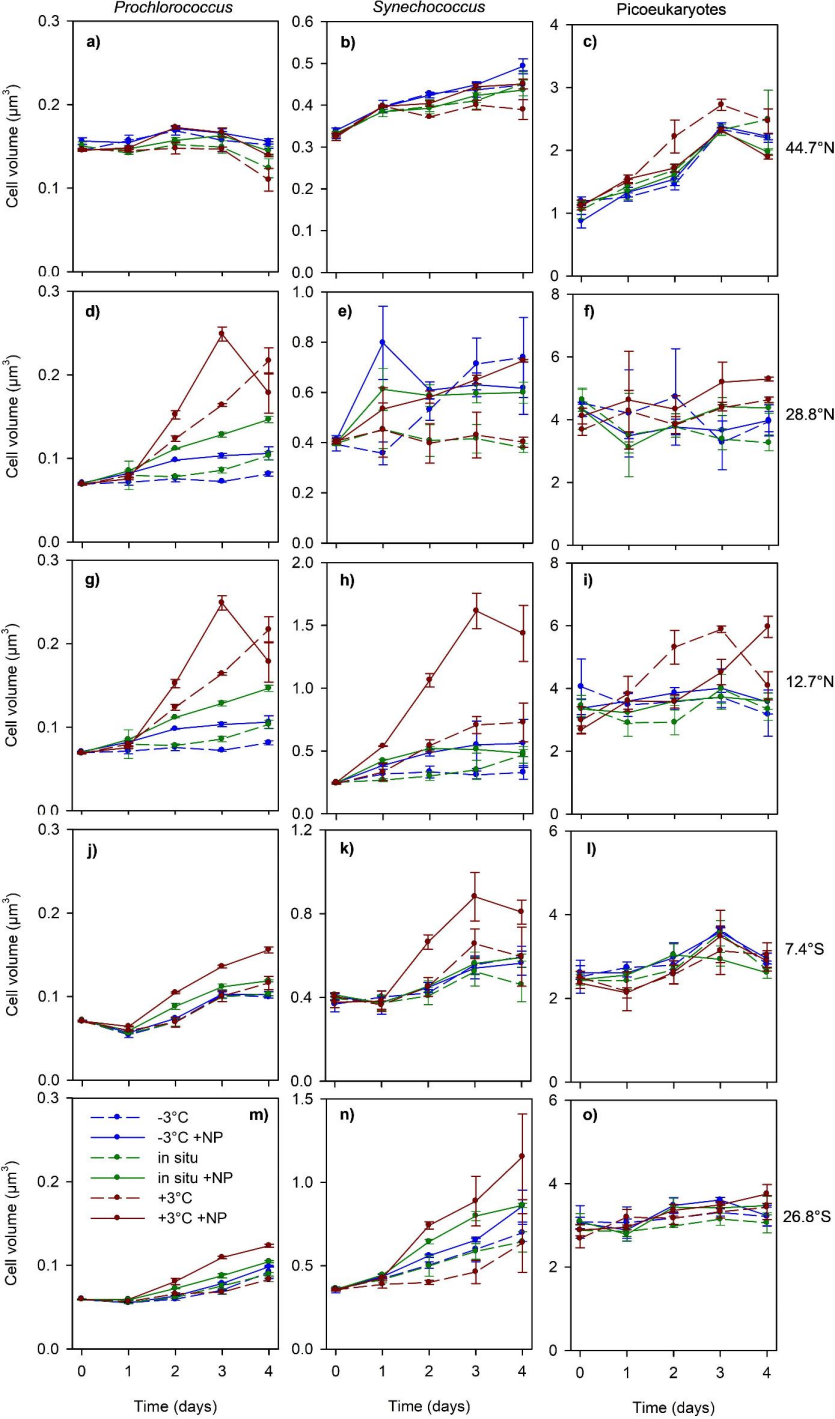
Evolution of the cell biovolume of *Prochlorococcus* (left panels), *Synechococcus* (middle panels) and picoeukaryotes (right panels) in the different treatments during each experiment. Vertical bars indicate standard deviation (SD).

We observed a general trend of increasing cell volume over time in most experiments and treatments, excluding *Prochlorococcus* at 44.7°N and picoeukaryotes at 28.8°N and 26.8°S (Fig. 2, Table S1). Aside from these exceptions, the cell volume tended to increase with incubation time (by up to 8-fold relative to initial values) under all combinations of temperature and nutrient availability. This increase in cell volume, as indicated by a significant value of the slope in the regression of cell volume versus time (Table S1), was particularly prevalent in the case of *Prochlorococcus* and *Synechococcus*. For *Prochlorococcus*, we found strong evidence for a cell volume increase in 24 out of 30 instances (5 experiments × 6 treatments), whereas in *Synechococcus* the same occurred in 26 out of 30 instances (Table S1). In contrast, evidence for an increase in the cell volume of picoeukaryotes was limited to 11 cases. While, on a few occasions, the greatest increases in cell volume occurred in the warmed treatments (especially + 3 °C + NP) (Fig. 2d, g,h, k), the increase in cell volume of a given picophytoplankton group was often similar across all the treatments (Fig. 2b, c,j, l,m). Considering all the experiments, treatments, sampling days and replicates (*n* = 450), the range of observed cell volumes was 5-fold for *Prochlorococcus* (0.05–0.25 μm^3^), 8-fold for *Synechococcus* (0.2–1.6 μm^3^) and 6-fold (1–6 μm^3^) for the picoeukaryotes.

### Effect of experimental treatments on cell volume

**Fig. 3:**
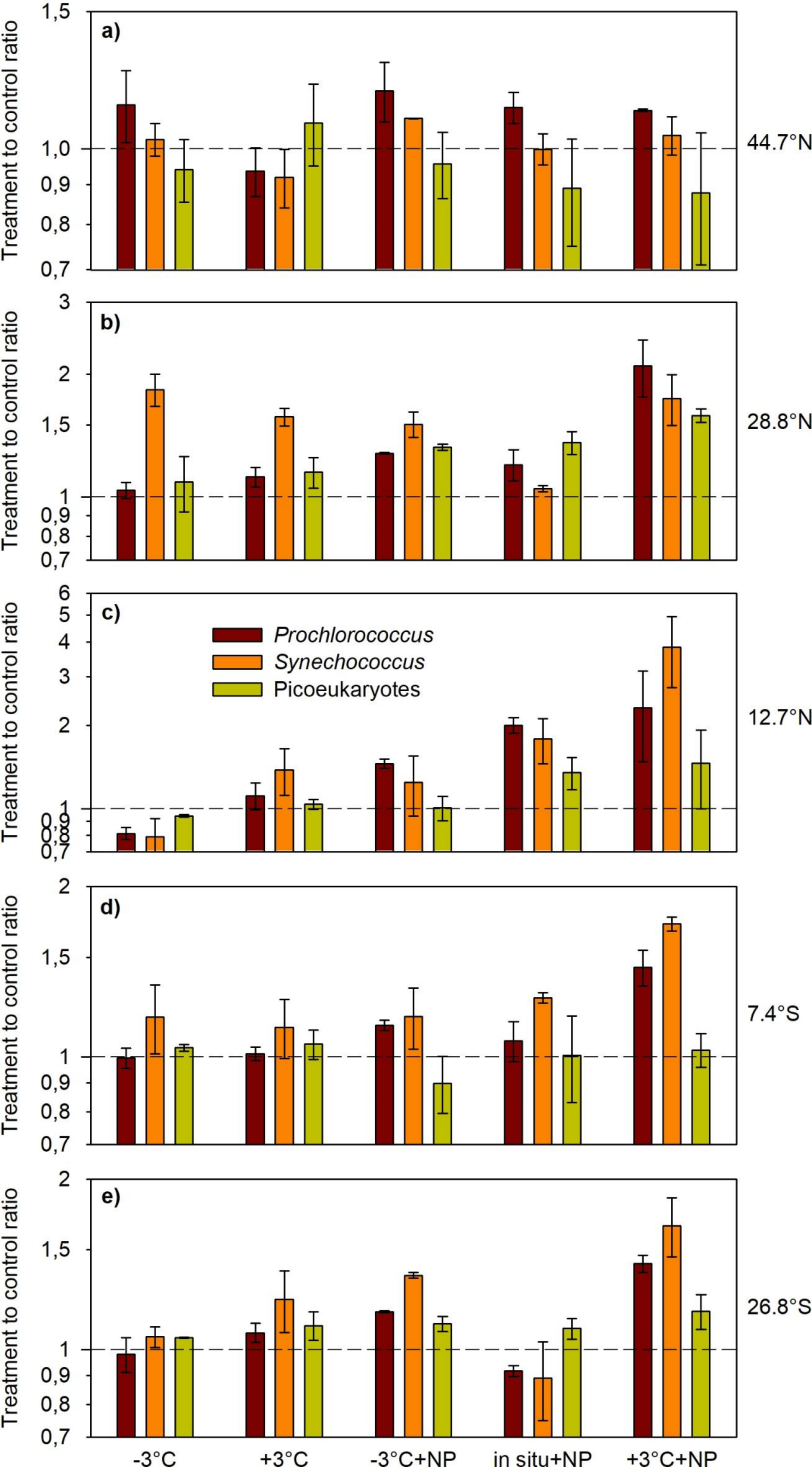
Effect of experimental treatments on the cell biovolume for each picophytoplankton group and experiment. The mean value of the ratio between the cell biovolume on each treatment and the cell biovolume in the control during days 3–4 of each experiment is shown. A value of 1 (horizontal dashed line) indicates no difference between cell biovolume in a treatment and cell volume in the control. Note the differences in the Y-axis scale. Vertical lines indicate SD.

We assessed the effect of each experimental treatment by calculating the treatment to control cell volume ratio on days 3–4 (Fig. 3). The incubation conducted at 44.7°N was the one in which the experimental treatments had the least effect on cell volume, as the differences between the treatments and the control were always small (< 15%) (Fig. 3a) and, in most cases, not significant (Table S2). In the other experiments, substantial (> 50%) changes in cell volume were found for some groups (most commonly, *Prochlorococcus* and *Synechococcus*) and/or experimental treatments, with the dominant response being an increase. At 28.8°N, the size of *Synechococcus* increased significantly in response to all treatments except − 3 °C + NP (Table S2). This was the only experiment in which the warming and cooling treatments (without nutrient addition) gave way to a change > 50% in the cell volume of any group. The combination of warming and nutrient addition (+ 3 °C + NP) caused the most pronounced response at 28.8°N, leading to significant increases in the cell volume of all three picophytoplankton groups (Table S2). The common results of the experiments conducted at 12.7°N, 7.4°S and 26.8°S were that (i) temperature as a single driver (treatments − 3 °C and + 3 °C) tended to have a small effect on the cell volume of all the groups and that (ii) the cell volume of both *Prochlorococcus* and *Synechococcus* increased significantly (Table S2) under warming and nutrient-enriched conditions (+ 3 °C + NP) (Fig. 3c, d,e). Nutrient enrichment alone (in situ + NP) also caused a significant increase in the cell volume of *Prochlorococcus* and *Synechococcus* at 12.7°N.

**Fig. 4:**
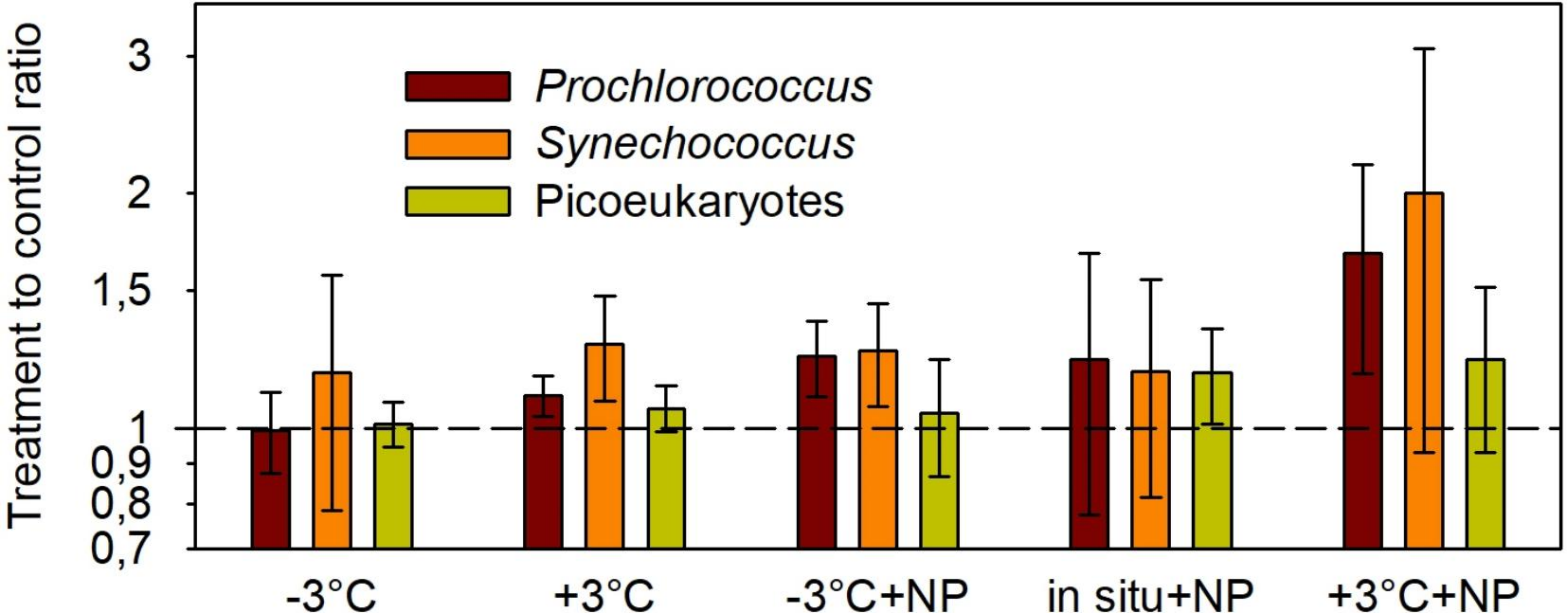
Overall effect of experimental treatment on the cell biovolume of each picophytoplankton group. The mean treatment to control biovolume ratio was calculated with data from all five experiments pooled together. A value of 1 (dashed line) indicates no difference between the cell biovolume in a treatment and the cell volume in the control. Vertical lines indicate SD.

The results of all five experiments together (Fig. 4) can be summarized as follows: (i) picophytoplankton cell volume tends to increase in response to changes in temperature and/or nutrient availability; (ii) picoeukaryote cell volume is less responsive than that of picocyanobacteria and increases moderately only under nutrient-enriched conditions; and (iii) *Prochlorococcus* and *Synechococcus* exhibit the most marked increase in their cell volume under conditions of warming and nutrient enrichment.

### Relationship between growth rate and changes in cell volume

**Fig. 5:**
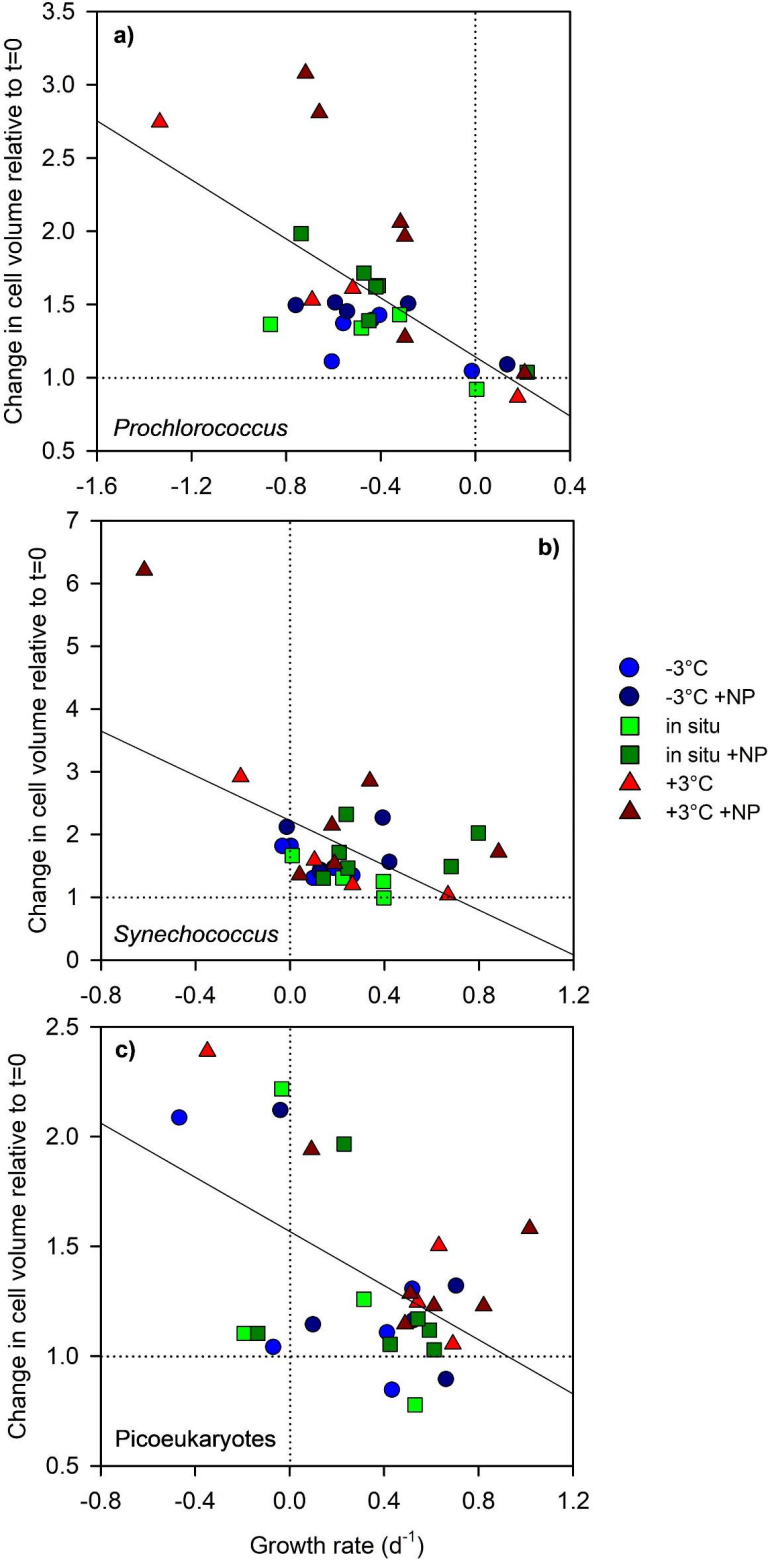
Relationship between population net growth rates and relative change in cell biovolume for (a) *Prochlorococcus*, (b) *Synechococcus* and (c) picoeukaryotes. Growth rates were calculated from the change in cell abundance between day 2 and day 4, whereas the change in cell biovolume was computed as the quotient between the mean cell biovolume in each treatment over the same period and the initial cell biovolume at t = 0. The dotted horizontal line indicates no change in the cell biovolume relative to the initial value. The dotted vertical line denotes a net growth rate of 0. The linear regression fits (shown as solid lines) were *y* = 1.14–1.00 *x* (*r*^2^ = 0.42, *n* = 30, *p* < 0.001), *y* = 2.22–1.78 *x* (*r*^2^ = 0.28, *n* = 30, *p* < 0.005) and *y* = 1.57–0.62 *x* (*r*^2^ = 0.24, *n* = 30, *p* < 0.005) for *Prochlorococcus*, *Synechococcus* and picoeukaryotes, respectively.

We found a general trend, in all three picophytoplankton groups, of greater increases in cell volume as the population growth rates decreased (Fig. 5). In *Synechococcus* and picoeukaryotes, the largest increases in cell volume (by approximately 2-fold or more) coincided with populations that had slow (< 0.4 d^− 1^) growth rates (Fig. 5b, c). In *Prochlorococcus*, which presented negative growth rates in most cases, increases in cell volume (relative to the initial values) were small in populations with growth rates between 0 and 0.3 d^− 1^, but took progressively larger values (> 1.5-fold) as growth rates became more negative (Fig. 5a).

### Abundance- versus biovolume-based growth rates

**Fig. 6:**
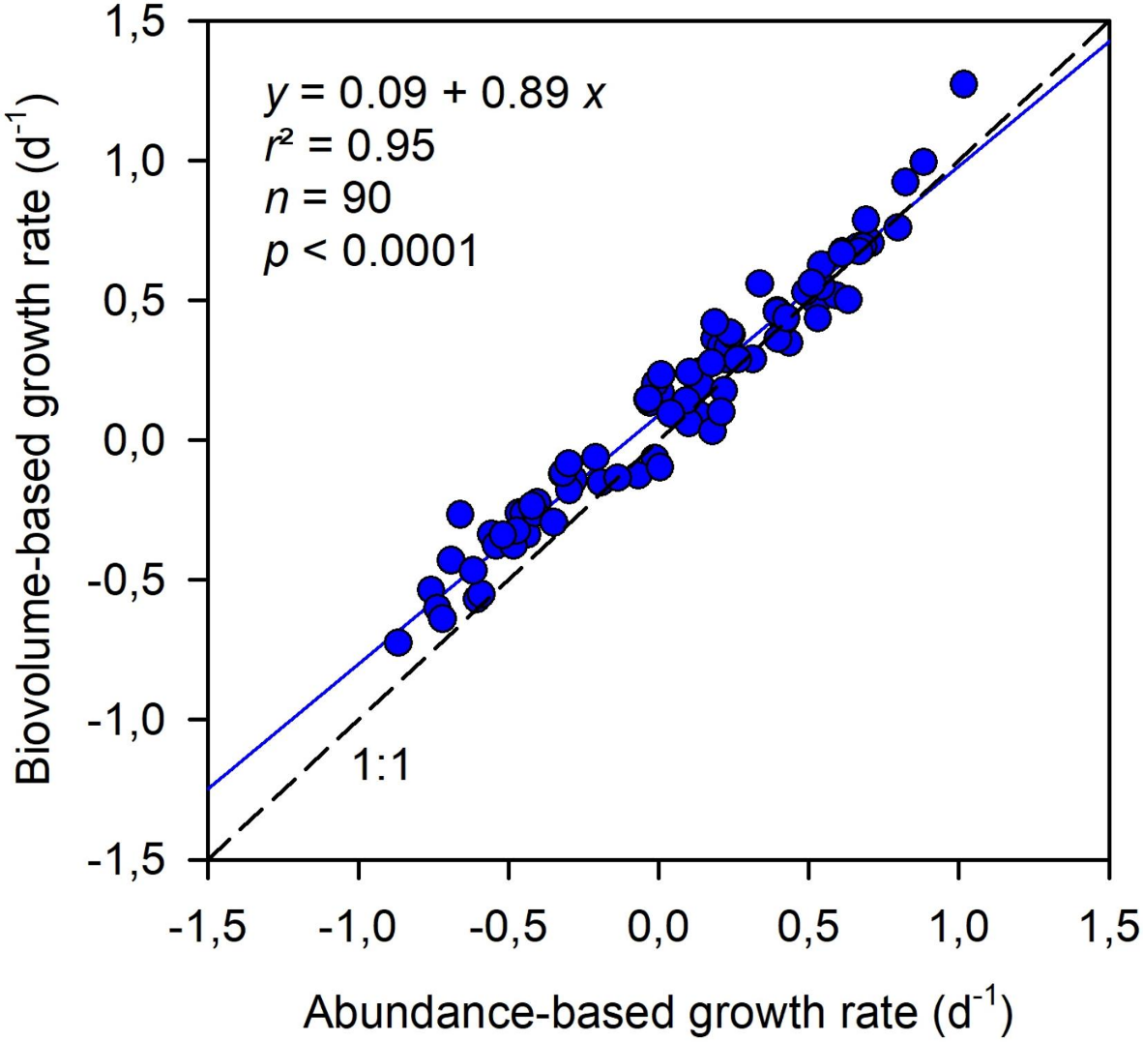
Relationship between the abundance-based and biovolume-based net growth rates for all the treatments and experiments combined. The linear regression equation with 95% confidence intervals is *y* = 0.09 [0.06, 0.11] + 0.89 [0.84, 0.93] *x* (*r*^2^ = 0.95, *n* = 90, *p* < 0.0001).

There was a very strong correlation between the abundance- and biovolume-based growth rates, but the slope of the linear relationship between these two metrics was 0.89, which was significantly different from 1 (95% confidence interval: 0.84, 0.93; Fig. 6). While the agreement between the abundance- and biovolume-based growth rates was very good for growth rates > 0.3 d^− 1^ (data close to the 1:1 line in Fig. 6), the two metrics differed for growth rates between < 0.3 d^− 1^, especially for negative growth rates. This occurred because, as shown above, negative growth rates are typically associated with increases in cell volume, which results in higher (or less negative) biovolume-based growth rates than abundance-based rates. For the subset of data pairs in which the abundance-based growth rates were negative, biovolume-based growth rates were higher (less negative) by a median value of 34% (SD = 20%).

## Discussion

On the basis on five experiments conducted under a wide range of environmental conditions over a large spatial scale, our observations reveal general trends in the variability of picophytoplankton cell size that are relevant for interpreting results from in vitro incubations and, in addition, shed light on potential mechanisms underlying changes in the mean cell size of phytoplankton populations in the sea.

A common trend in our experiments was the increase in the cell size of *Prochlorococcus* and *Synechococcus* over time. Given that this increase occurred in all experimental treatments and also in the control, it is likely that it was a result of sample confinement itself rather than any particular combination of growth conditions. One possibility is that bottle enclosure may have given way to trophic cascades^42^, whereby reduced abundances of heterotrophic large nanoplankton and microplankton lead to increased abundances of smaller bacterivore and mixotrophic flagellates and therefore enhanced grazing pressure on picocyanobacteria^43,44^. If the inverse relationship between cell size and susceptibility to grazing holds^17,45–47^, the increased grazing pressure on *Prochlorococcus* and *Synechococcus* may have selected for larger cells. Another possibility is the occurrence of toxicity effects inside the incubation bottles, but this is unlikely because in all experiments there were clear positive responses to nutrient enrichment, as indicated by increases in total chlorophyll *a* concentration, picophytoplankton abundance and cell-specific chlorophyll fluorescence in all picophytoplankton groups^38^. It must be noted that the existence of bottle effects does not invalidate the conclusions based on differences between control and treatments, as has been demonstrated for the role of iron in controlling phytoplankton stocks and growth rates in high-nutrient low-chlorophyll regions^48,49^ and more generally for the identification of global patterns of nutrient limitation and co-limitation across the ocean^50^. Our results, however, do support the use of biovolume concentration instead of abundance and constant carbon conversion factors to obtain more accurate assessments of phytoplankton standing stocks, because variability in population mean cell size can be substantial (> 6-fold), particularly in the case of *Prochlorococcus* and *Synechococcus*.

According to the temperature-size rule in ectotherms, there is an inverse relationship between temperature and body size^51^. In the case of aquatic protists, an analysis of laboratory data indicates that on average cell size decreases with increasing temperature at a rate of approximately 2.5% per °C (ref. 52), which in our experiments could have resulted in a 15% range of variability, since the total range of temperatures assayed spanned 6 °C. However, we did not find clear evidence of temperature effects on cell size, and in fact the dominant response to the + 3 °C treatment was an increase in cell volume, instead of a decrease. This may have resulted from the short duration of the incubations preventing a full phenotypic acclimation response, and from the fact that we measured only the mean population cell size, instead of the size of cells within a single growth state (for example, recently divided cells)^52^. An additional explanation is that changes in cell size with temperature are mediated by thermal effects on the metabolic rate and resource requirements, as well as changes in the growth rate^53^. However, the temperature sensitivity of phytoplankton metabolism and growth is greatly reduced under conditions of nutrient limitation^36,54,55^. The above-mentioned rate of cell size decrease of 2.5% °C^− 1^ originates from laboratory cultures maintained under resource-sufficient conditions, and therefore represents an upper limit for the temperature sensitivity of cell size that is unlikely to be realized under oligotrophic conditions. To the extent that temperature effects on cell size are related to changes in metabolic rates and growth, the expectation that phytoplankton cells will become smaller in a warming ocean^56,57^ must be tempered by the fact that under nutrient limiting conditions, which prevail over most of the ocean^50,58^, the direct physiological effects of temperature are attenuated^38,59–62^.

The most consistent effect of an experimental treatment in our incubations was the increase in the cell size of *Prochlorococcus* and *Synechococcus* under warming and nutrient-enriched conditions. This treatment was the one in which the greatest increases in picophytoplankton standing stocks and light-harvesting capacity were observed, and where the picoeukaryotes appeared to be favored to the detriment of *Prochlorococcus* and *Synechococcus*^38^. The increased cell size of the picocyanobacteria may have resulted from a decrease in their intrinsic growth rates, as they tend to be outcompeted by picoeukaryotes under conditions of increased nutrient availability^21,22,63^. In addition, it is possible that microzooplankton consumption of *Prochlorococcus* and *Synechococcus* was stimulated by warming^64,65^, with increased predation resulting in the selection of larger cell sizes as a result of the inverse relationship between picocyanobacterial cell size and vulnerability to grazing^45,46^.

We found, in all three picophytoplankton groups, an inverse relationship between growth rate and cell size that appeared to be independent of the specific conditions leading to fast or slow growth. This result supports the conclusions of previous laboratory studies with batch cultures of prokaryotic and eukaryotic phytoplankton, which reported an increase in the mean cell size when populations reach the end of the exponential growth phase and enter the stationary phase^37–40^. The increase in cell volume as cells enter the stationary phase likely arises from the ability of cells to carry on fixing carbon photosynthetically even though nutrient limitation slows down cell division^66^. The inverse relationship between growth rate and cell volume has also been demonstrated through cell-cycle analysis of light-limited cultures of *Prochlorococcus* and *Synechococcus*, where the durations of the pre- and post-DNA replication periods both decreased with increasing growth rate^67^. With respect to field observations, an inverse relationship between growth rate and cell volume has been found through the analysis of seasonal cycles of variability in *Synechococcus* properties in a coastal temperate ecosystem^30^. Thus, in picophytoplankton, and probably in phytoplankton in general, conditions that induce slow growth lead to increased mean population cell sizes. The vertical pattern of increasing cell size of *Prochlorococcus* and *Synechococcus* with increasing depth^1,23,24,26,31^ can therefore be attributed to the decreased growth rates that are often found near the base of the euphotic layer^68–71^, which probably result from a combination of light and temperature limitations.

In conclusion, we have shown that the mean cell size of picophytoplankton populations can display a large degree of variability, which presumably would have been even greater if samples from different depths in the euphotic layer had been considered. These results emphasize the need to consider changes in cell volume when assessing the dynamics of picophytoplankton standing stocks and calculating rates of biomass production. The dominant response of cell volume to confinement and to the various combinations of temperature and nutrient availability was an increase, which was more pronounced in *Prochlorococcus* and *Synechococcus* than in the picoeukaryotes. We observed a trend toward larger mean cell sizes in populations experiencing low and negative growth rates. The inverse relationship between growth rate and mean population cell size appears to be a general pattern in phytoplankton that is observed both in cultures and natural assemblages irrespective of the specific growth-limiting factor.

## Electronic supplementary material

Below is the link to the electronic supplementary material.

Supplementary Material 1

## Data Availability

The datasets used during the current study are available from the corresponding author upon reasonable request.
