# Supplementary material for: Effect of temperature, nutrients and growth rate on picophytoplankton cell size across the Atlantic Ocean
Source: Sci Rep. 2024 Nov 14;14:28034. doi: 10.1038/s41598-024-78951-w (PMC11564571; doi:10.1038/s41598-024-78951-w)
Supplement: Supplementary file 1 — Supplementary Material 1 [file 41598_2024_78951_MOESM1_ESM.docx]

**Supplementary Information**

**Effect of temperature, nutrients and growth rate on picophytoplankton cell size across the Atlantic Ocean**

Emilio Marañón, Cristina Fernández-González, Glen A. Tarran

**Supplementary Figure 1.** Example size-fractionation plot for estimating the median cell size of *Synechococcus* (SYN), *Prochlorococcus* (PRO) and picoeukaryotes (PICOEUK). The plot shows the relationship between filter pore size (µm) and the percentage of cells remaining in the filtrate for each picophytoplankton group. The resulting median cell diameter is illustrated for *Synechococcus*. Water taken from 100 m (deep chlorophyll maximum), 21 October 2019.

**Supplementary Table 1.** Results of the linear regression analysis of cell volume (*y*) as a function of sampling day (*x*) for *Prochlorococcus* (Pro), *Synechococcus* (Syn) and picoeukaryotes (Peuka) in each experiment and treatment. The linear regression model is *y* = *a* + *b* *x*. In all cases, *n* = 15.

| **Experiment** | **Group** | **Treatment** | ***a*** | ***b*** | ***r*^2^** | ***p*** |
| --- | --- | --- | --- | --- | --- | --- |
| 44.7°N | Pro | −3°C | 0.15 | 0.001 | 0.03 | 0.50 |
| 44.7°N | Syn | −3°C | 0.35 | 0.028 | 0.74 | <0.0001 |
| 44.7°N | Peuka | −3°C | 1.07 | 0.302 | 0.81 | <0.0001 |
| 44.7°N | Pro | −3°C +NP | 0.16 | 0.001 | 0.05 | 0.43 |
| 44.7°N | Syn | −3°C +NP | 0.35 | 0.036 | 0.95 | <0.0001 |
| 44.7°N | Peuka | −3°C +NP | 0.92 | 0.374 | 0.88 | <0.0001 |
| 44.7°N | Pro | in situ | 0.15 | -0.004 | 0.30 | 0.03 |
| 44.7°N | Syn | in situ | 0.34 | 0.027 | 0.82 | <0.0001 |
| 44.7°N | Peuka | in situ | 1.04 | 0.377 | 0.87 | <0.0001 |
| 44.7°N | Pro | in situ +NP | 0.15 | 0.001 | 0.06 | 0.38 |
| 44.7°N | Syn | in situ +NP | 0.34 | 0.024 | 0.84 | <0.0001 |
| 44.7°N | Peuka | in situ +NP | 1.16 | 0.25 | 0.75 | <0.0001 |
| 44.7°N | Pro | +3°C | 0.15 | -0.007 | 0.41 | 0.012 |
| 44.7°N | Syn | +3°C | 0.35 | 0.012 | 0.39 | 0.016 |
| 44.7°N | Peuka | +3°C | 1.22 | 0.39 | 0.81 | <0.0001 |
| 44.7°N | Pro | +3°C +NP | 0.15 | 0.001 | 0.00 | 0.81 |
| 44.7°N | Syn | +3°C +NP | 0.34 | 0.030 | 0.86 | <0.0001 |
| 44.7°N | Peuka | +3°C +NP | 1.25 | 0.23 | 0.68 | <0.0001 |
| 28.8°N | Pro | −3°C | 0.07 | 0.007 | 0.71 | <0.0001 |
| 28.8°N | Syn | −3°C | 0.34 | 0.104 | 0.72 | <0.0001 |
| 28.8°N | Peuka | −3°C | 4.55 | -0.211 | 0.09 | 0.26 |
| 28.8°N | Pro | −3°C +NP | 0.07 | 0.011 | 0.94 | <0.0001 |
| 28.8°N | Syn | −3°C +NP | 0.56 | 0.024 | 0.06 | 0.38 |
| 28.8°N | Peuka | −3°C +NP | 3.95 | -0.055 | 0.04 | 0.47 |
| 28.8°N | Pro | in situ | 0.07 | 0.007 | 0.55 | 0.0015 |
| 28.8°N | Syn | in situ | 0.42 | -0.007 | 0.04 | 0.45 |
| 28.8°N | Peuka | in situ | 4.28 | -0.28 | 0.31 | 0.035 |
| 28.8°N | Pro | in situ +NP | 0.07 | 0.015 | 0.91 | <0.0001 |
| 28.8°N | Syn | in situ +NP | 0.48 | 0.038 | 0.36 | 0.017 |
| 28.8°N | Peuka | in situ +NP | 3.74 | 0.138 | 0.11 | 0.21 |
| 28.8°N | Pro | +3°C | 0.07 | 0.014 | 0.75 | <0.0001 |
| 28.8°N | Syn | +3°C | 0.42 | -0.002 | 0.00 | 0.81 |
| 28.8°N | Peuka | +3°C | 3.75 | 0.199 | 0.40 | 0.011 |
| 28.8°N | Pro | +3°C +NP | 0.05 | 0.041 | 0.91 | <0.0001 |
| 28.8°N | Syn | +3°C +NP | 0.42 | 0.077 | 0.89 | <0.0001 |
| 28.8°N | Peuka | +3°C +NP | 4.13 | 0.293 | 0.27 | 0.045 |
| 12.7°N | Pro | −3°C | 0.07 | 0.002 | 0.57 | 0.001 |
| 12.7°N | Syn | −3°C | 0.27 | 0.016 | 0.26 | 0.052 |
| 12.7°N | Peuka | −3°C | 3.90 | -0.152 | 0.16 | 0.132 |
| 12.7°N | Pro | −3°C +NP | 0.07 | 0.009 | 0.88 | <0.0001 |
| 12.7°N | Syn | −3°C +NP | 0.29 | 0.079 | 0.53 | 0.002 |
| 12.7°N | Peuka | −3°C +NP | 3.52 | 0.080 | 0.09 | 0.28 |
| 12.7°N | Pro | in situ | 0.07 | 0.008 | 0.64 | <0.001 |
| 12.7°N | Syn | in situ | 0.22 | 0.052 | 0.70 | <0.0001 |
| 12.7°N | Peuka | in situ | 3.15 | 0.085 | 0.05 | 0.39 |
| 12.7°N | Pro | in situ +NP | 0.07 | 0.019 | 0.99 | <0.0001 |
| 12.7°N | Syn | in situ +NP | 0.32 | 0.056 | 0.60 | <0.001 |
| 12.7°N | Peuka | in situ +NP | 3.30 | 0.098 | 0.21 | 0.08 |
| 12.7°N | Pro | +3°C | 0.05 | 0.038 | 0.95 | <0.0001 |
| 12.7°N | Syn | +3°C | 0.24 | 0.134 | 0.84 | <0.0001 |
| 12.7°N | Peuka | +3°C | 3.57 | 0.425 | 0.29 | 0.036 |
| 12.7°N | Pro | +3°C +NP | 0.07 | 0.039 | 0.66 | <0.001 |
| 12.7°N | Syn | +3°C +NP | 0.29 | 0.345 | 0.85 | <0.0001 |
| 12.7°N | Peuka | +3°C +NP | 2.57 | 0.74 | 0.87 | <0.0001 |
| 7.4°S | Pro | −3°C | 0.06 | 0.010 | 0.62 | <0.001 |
| 7.4°S | Syn | −3°C | 0.06 | 0.346 | 0.83 | <0.0001 |
| 7.4°S | Peuka | −3°C | 0.15 | 0.071 | 0.26 | 0.053 |
| 7.4°S | Pro | −3°C +NP | 0.01 | 0.060 | 0.68 | <0.001 |
| 7.4°S | Syn | −3°C +NP | 0.37 | 0.048 | 0.69 | <0.001 |
| 7.4°S | Peuka | −3°C +NP | 2.61 | 0.164 | 0.36 | 0.018 |
| 7.4°S | Pro | in situ | 0.06 | 0.011 | 0.68 | <0.001 |
| 7.4°S | Syn | in situ | 0.37 | 0.028 | 0.32 | 0.027 |
| 7.4°S | Peuka | in situ | 2.42 | 0.164 | 0.25 | 0.055 |
| 7.4°S | Pro | in situ +NP | 0.06 | 0.014 | 0.83 | <0.0001 |
| 7.4°S | Syn | in situ +NP | 0.37 | 0.054 | 0.75 | <0.0001 |
| 7.4°S | Peuka | in situ +NP | 2.58 | 0.070 | 0.14 | 0.174 |
| 7.4°S | Pro | +3°C | 0.06 | 0.013 | 0.73 | <0.0001 |
| 7.4°S | Syn | +3°C | 0.35 | 0.067 | 0.55 | 0.001 |
| 7.4°S | Peuka | +3°C | 2.27 | 0.205 | 0.38 | 0.014 |
| 7.4°S | Pro | +3°C +NP | 0.06 | 0.024 | 0.91 | <0.0001 |
| 7.4°S | Syn | +3°C +NP | 0.35 | 0.136 | 0.79 | <0.0001 |
| 7.4°S | Peuka | +3°C +NP | 2.21 | 0.24 | 0.40 | 0.011 |
| 26.8°S | Pro | −3°C | 0.05 | 0.008 | 0.68 | <0.001 |
| 26.8°S | Syn | −3°C | 0.34 | 0.085 | 0.92 | <0.0001 |
| 26.8°S | Peuka | −3°C | 3.07 | 0.050 | 0.08 | 0.289 |
| 26.8°S | Pro | −3°C +NP | 0.05 | 0.010 | 0.82 | <0.0001 |
| 26.8°S | Syn | −3°C +NP | 0.33 | 0.121 | 0.93 | <0.0001 |
| 26.8°S | Peuka | −3°C +NP | 3.03 | 0.107 | 0.24 | 0.060 |
| 26.8°S | Pro | in situ | 0.052 | 0.008 | 0.81 | <0.0001 |
| 26.8°S | Syn | in situ | 0.35 | 0.072 | 0.88 | <0.0001 |
| 26.8°S | Peuka | in situ | 2.85 | 0.066 | 0.32 | 0.026 |
| 26.8°S | Pro | in situ +NP | 0.05 | 0.012 | 0.92 | <0.0001 |
| 26.8°S | Syn | in situ +NP | 0.35 | 0.136 | 0.97 | <0.0001 |
| 26.8°S | Peuka | in situ +NP | 2.96 | 0.139 | 0.42 | 0.008 |
| 26.8°S | Pro | +3°C | 0.05 | 0.006 | 0.79 | <0.0001 |
| 26.8°S | Syn | +3°C | 0.32 | 0.063 | 0.54 | 0.002 |
| 26.8°S | Peuka | +3°C | 2.83 | 0.161 | 0.56 | 0.001 |
| 26.8°S | Pro | +3°C +NP | 0.05 | 0.017 | 0.93 | <0.0001 |
| 26.8°S | Syn | +3°C +NP | 0.30 | 0.205 | 0.86 | <0.0001 |
| 26.8°S | Peuka | +3°C +NP | 2.83 | 0.226 | 0.85 | <0.0001 |

**Supplementary Table 2.** Results of the two-tailed Student’s t-test conducted to compare the log-transformed mean cell sizes of *Prochlorococcus* (Pro), *Synechococcus* (Syn) and picoeukaryotes (Peuka) between each treatment and the control at the end of each experiment.

| **Experiment** | **Group** | **Treatment** | **t(4)** | ***p* value** |
| --- | --- | --- | --- | --- |
| 44.7°N | Pro | −3°C | 4.02 | 0.027 |
| 44.7°N | Pro | −3°C +NP | 4.77 | 0.041 |
| 44.7°N | Pro | in situ +NP | 2.87 | 0.064 |
| 44.7°N | Pro | +3°C | -1.35 | 0.246 |
| 44.7°N | Pro | +3°C +NP | 2.34 | 0.144 |
| 44.7°N | Syn | −3°C | -0.11 | 0.917 |
| 44.7°N | Syn | −3°C +NP | 2.17 | 0.119 |
| 44.7°N | Syn | in situ +NP | -0.69 | 0.524 |
| 44.7°N | Syn | +3°C | -2.88 | 0.044 |
| 44.7°N | Syn | +3°C +NP | -0.01 | 0.989 |
| 44.7°N | Peuka | −3°C | -1.10 | 0.386 |
| 44.7°N | Peuka | −3°C +NP | -1.00 | 0.419 |
| 44.7°N | Peuka | in situ +NP | -1.93 | 0.192 |
| 44.7°N | Peuka | +3°C | -0.09 | 0.929 |
| 44.7°N | Peuka | +3°C +NP | -2.23 | 0.155 |
| 28.8°N | Pro | −3°C | 0.04 | 0.970 |
| 28.8°N | Pro | −3°C +NP | 1.78 | 0.216 |
| 28.8°N | Pro | in situ +NP | 2.59 | 0.060 |
| 28.8°N | Pro | +3°C | 2.00 | 0.114 |
| 28.8°N | Pro | +3°C +NP | 11.04 | 0.000 |
| 28.8°N | Syn | −3°C | 3.88 | 0.060 |
| 28.8°N | Syn | −3°C +NP | 3.89 | 0.060 |
| 28.8°N | Syn | in situ +NP | 8.34 | 0.003 |
| 28.8°N | Syn | +3°C | 1.46 | 0.216 |
| 28.8°N | Syn | +3°C +NP | 32.45 | 0.000 |
| 28.8°N | Peuka | −3°C | 2.87 | 0.045 |
| 28.8°N | Peuka | −3°C +NP | 2.22 | 0.112 |
| 28.8°N | Peuka | in situ +NP | 6.36 | 0.007 |
| 28.8°N | Peuka | +3°C | 8.78 | 0.012 |
| 28.8°N | Peuka | +3°C +NP | 13.44 | 0.005 |
| 12.7°N | Pro | −3°C | -7.22 | 0.005 |
| 12.7°N | Pro | −3°C +NP | 0.48 | 0.662 |
| 12.7°N | Pro | in situ +NP | 10.96 | 0.002 |
| 12.7°N | Pro | +3°C | 14.68 | 0.000 |
| 12.7°N | Pro | +3°C +NP | 6.21 | 0.024 |
| 12.7°N | Syn | −3°C | -2.88 | 0.044 |
| 12.7°N | Syn | −3°C +NP | 0.68 | 0.541 |
| 12.7°N | Syn | in situ +NP | 0.38 | 0.729 |
| 12.7°N | Syn | +3°C | 2.74 | 0.071 |
| 12.7°N | Syn | +3°C +NP | 9.25 | 0.001 |
| 12.7°N | Peuka | −3°C | -0.44 | 0.689 |
| 12.7°N | Peuka | −3°C +NP | 0.76 | 0.487 |
| 12.7°N | Peuka | in situ +NP | 1.04 | 0.355 |
| 12.7°N | Peuka | +3°C | 2.26 | 0.086 |
| 12.7°N | Peuka | +3°C +NP | 2.35 | 0.003 |
| 7.4°S | Pro | −3°C | -2.18 | 0.117 |
| 7.4°S | Pro | −3°C +NP | -0.29 | 0.787 |
| 7.4°S | Pro | in situ +NP | 10.04 | 0.002 |
| 7.4°S | Pro | +3°C | 2.88 | 0.101 |
| 7.4°S | Pro | +3°C +NP | 21.9 | 0.000 |
| 7.4°S | Syn | −3°C | 2.29 | 0.105 |
| 7.4°S | Syn | −3°C +NP | 1.73 | 0.182 |
| 7.4°S | Syn | in situ +NP | 2.43 | 0.134 |
| 7.4°S | Syn | +3°C | 1.44 | 0.223 |
| 7.4°S | Syn | +3°C +NP | 5.05 | 0.014 |
| 7.4°S | Peuka | −3°C | 2.69 | 0.073 |
| 7.4°S | Peuka | −3°C +NP | 3.52 | 0.072 |
| 7.4°S | Peuka | in situ +NP | -0.94 | 0.444 |
| 7.4°S | Peuka | +3°C | 2.19 | 0.160 |
| 7.4°S | Peuka | +3°C +NP | 1.51 | 0.270 |
| 26.8°S | Pro | −3°C | 0.52 | 0.655 |
| 26.8°S | Pro | −3°C +NP | 7.25 | 0.018 |
| 26.8°S | Pro | in situ +NP | 10.8 | 0.002 |
| 26.8°S | Pro | +3°C | -2.96 | 0.059 |
| 26.8°S | Pro | +3°C +NP | 20.9 | 0.000 |
| 26.8°S | Syn | −3°C | 1.19 | 0.299 |
| 26.8°S | Syn | −3°C +NP | 3.37 | 0.027 |
| 26.8°S | Syn | in situ +NP | 5.42 | 0.032 |
| 26.8°S | Syn | +3°C | -0.19 | 0.862 |
| 26.8°S | Syn | +3°C +NP | 4.26 | 0.023 |
| 26.8°S | Peuka | −3°C | 0.78 | 0.477 |
| 26.8°S | Peuka | −3°C +NP | 0.88 | 0.427 |
| 26.8°S | Peuka | in situ +NP | 2.18 | 0.095 |
| 26.8°S | Peuka | +3°C | 1.69 | 0.165 |
| 26.8°S | Peuka | +3°C +NP | 3.62 | 0.022 |
